# Transferability and Reproducibility of the HepaRG CometChip Assay

**DOI:** 10.1002/em.70037

**Published:** 2025-11-26

**Authors:** Leslie Recio, Carol Swartz, Lincoln Martin, Emily Rottinger, Stefan Pfuhler, Erica Pinkus, Daniel J. Roberts, Leon F. Stankowski, Simran Kaushal, Norah A. Owiti, Bevin P. Engelward

**Affiliations:** ^1^ Integrated Laboratory Systems Research Triangle Park North Carolina USA; ^2^ ScitoVation Durham North Carolina USA; ^3^ Inotiv RTP Morrisville North Carolina USA; ^4^ Proctor & Gamble Co. Cincinnati Ohio USA; ^5^ Charles River Laboratories Illinois USA; ^6^ Toxys Inc. New York New York USA; ^7^ Department of Biological Engineering Massachusetts Institute of Technology Cambridge Massachusetts USA

**Keywords:** alkaline comet, comet assay, CometChip, genotoxicity testing, HepaRG

## Abstract

This interlaboratory evaluation of HepaRG CometChip was conducted to assess transferability and reproducibility of this new approach methodology (NAM) across four laboratories. Concentrations inducing up to ~70% relative cytotoxicity were determined by the organizing laboratory, and frozen chemical formulation blocks were sent to each participant. When noncytotoxic, 10 mM was the maximum dose. Cultures were exposed once daily for three consecutive days, and both cytotoxicity assessment, via ATP quantification, and comet analysis, commenced 3–4 h after initiation of the final exposure. Positive response was statistical pairwise significance (*p* < 0.05) with concentration‐related increases in %Tail DNA across ≥ 2 consecutive exposures. For 8 of 11 compounds, all four labs generated unanimous test results, with four negative compounds (2‐acetylaminofluorene [2‐AAF], 2,4‐dichlorophenol, eugenol and hydroquinone) and four positive compounds (azidothymidine, benzo(a)pyrene [BP], cyclophosphamide [CP], ethyl methanesulfonate).For the remaining chemicals, three of four labs generated negative calls for amitrole, cadmium chloride, and DMBA. In cases where bulky lesions were anticipated, the magnitude of %Tail DNA was low, due to the inherent insensitivity of the alkaline comet assay (not the CometChip per se) to detect bulky adducts repaired by nucleotide excision repair. This is supported by the small magnitude in %Tail DNA induced by BP and CP. Taken together, for all compounds there was majority agreement in CometChip results across participating laboratories supporting that the endpoint is readily transferable to new labs. Overall, this platform is a promising human‐relevant NAM, with a physiologically relevant detoxification process that could be incorporated into rodent replacement strategies.

## 1 Introduction

There are over 350,000 chemicals in use today with more than 2000 new chemical entities developed each year (Wang et al. 2020). For the vast majority there is insufficient knowledge of their toxicological risk to humans. This is a daunting challenge for toxicologists as existing testing paradigms do not have the required throughput to evaluate potential hazards and conduct appropriate risk assessments in a reasonable amount of time (Devito et al. 2024). In addition to chemicals, safety assessment is also an intricate part of pharmaceutical, fragrance, flavor, and medical device (among others) development. Collectively, xenobiotic exposures can have a wide range of biological consequences, including genotoxicity which can initiate cancer and promote other diseases.

Existing in vivo toxicity assays are not perfect, with animal‐to‐animal variability, limited throughput, and uncertainties regarding the biological alignment of responses in rodents to those that would be expected in humans (Andersen et al. 2019; Karmaus et al. 2022; Sewell et al. 2024). Furthermore, there are animal welfare concerns that have shaped regulatory policy, with animal testing of cosmetics having been banned in Europe for over a decade. A consequence of this growing societal pressure is that safety decisions become challenging for regulatory agencies charged with evaluating potential human health impacts if new tools are not available (and qualified) to replace the old. Hence the need for faster, more human‐relevant toxicity testing alternatives, which are collectively referred to as new approach methodologies (NAMs).

In response to concerns surrounding animal use in toxicology, researchers are developing human cell‐, organoid‐ and tissue‐based test systems that can be used across several industries to contribute to safety and risk assessments of environmental chemicals (Andersen et al. 2019; Harrill, Carstens, et al. 2024; Stucki et al. 2022). The ongoing revolution in the practice of toxicology has led to the development of new approach methodologies (NAMs) that incorporate human‐relevant “fit for purpose” in vitro assays that inform hazard and risk assessment decisions without the use of animal testing (Andersen et al. 2019; Harrill, Everett, et al. 2024; National Academies of Sciences, Engineering, and Medicine 2022, 2023; Stucki et al. 2022). Human‐relevant cell‐based NAMs can go beyond hazard identification to enable prediction of potential human risk (Moreau et al. 2022; Najjar et al. 2022; Paudel et al. 2023). However, the replacement of traditional animal genotoxicity tests with NAMs requires qualification of each test system. Technology needs to be transferred from the developers to external laboratories so that interlaboratory testing can commence to assess reproducibility and assay performance. Demonstrating that NAMs accurately predict both the responses known to occur in traditional regulatory testing, and more importantly what can occur in humans, requires extensive validation across multiple laboratories.

A significant limitation of many cell‐based assays is their deficiency in key xenobiotic metabolizing Phase I and Phase II enzymes. Regulatory cell‐based systems used in genetic toxicology do not have metabolic capacity and rely upon exogenous induced rat liver S9 fractions for bioactivation. Standard S9 mixes do not include co‐factors for Phase II enzymes, which can lead to false positives due to excessive formation of reactive Phase I metabolites. Conversely, HepaRG cell cultures do contain these important detoxifying enzymes (Recio et al. 2023). Furthermore, we have focused on human liver‐derived hepatocytes since the liver is the most frequent target organ observed in chronic cancer bioassays among more than 500 environmental chemicals tested by the National Toxicology Program (Shao et al. 2019; Guo and van den Beucken 2024).

HepaRG cells are emerging as a versatile cell type that is useful for in vitro drug metabolism studies, enzyme induction studies, in vitro toxicity testing, activation of nuclear receptors associated with non‐genotoxic liver carcinogens, and genotoxicity testing (Lübberstedt et al. 2011; Gutmann et al. 2023; Guo et al. 2024; Guo and van den Beucken 2024; Štampar and Žegura 2024). HepaRG cells have a strong functional resemblance to primary hepatocytes with respect to the spectrum of human Phase I CYP450 enzymes and Phase II (glucuronyltransferases, GSH‐transferases, sulfotransferases) drug metabolizing enzymes (Ramaiahgari et al. 2017; Franzosa et al. 2021). Furthermore, HepaRG hepatocytes have redundant biochemical and biological defenses that protect the genome from endogenous oxidative DNA damage. These include intracellular compartmentalization, overlapping enzymatic and nonenzymatic processes that detoxify ROS (e.g., superoxide dismutases, catalase/peroxidases, glutathione peroxidases), and intracellular antioxidants (e.g., glutathione) (Cho et al. 2022; Allameh et al. 2023). Given that many cell lines used for regulatory testing (and in high‐throughput testing platforms) lack xenobiotic metabolizing capabilities, there is a clear and pressing need to develop and validate metabolically competent human cell‐based assays. Metabolically competent HepaRG cells have already been used by several laboratories for several modalities to assess genotoxicity (Guo et al. 2024; Štampar and Žegura 2024), making them ideal cells for a higher‐throughput genotoxicity assay, as described below.

A classic approach for measuring DNA damage is the comet assay wherein the level of DNA damage can be measured by the extent to which DNA migrates through agarose. Undamaged DNA is highly supercoiled and resistant to migration, whereas damaged DNA that harbors strand breaks can lose superhelical tension, allowing loops of DNA to migrate away from the nucleoid, enabling quantification of %Tail DNA as a measure of DNA damage. While the traditional comet assay is slow, variable, and laborious, the CometChip (Wood et al. 2010) enables application of a 96‐well format and places the cells on the same focal plane so that a single image is sufficient to capture over 100 comets (Figure S1). Furthermore, software has been developed for objective automated analysis. As a result, the CometChip is orders of magnitude faster than the traditional comet assay, making it an attractive assay that has been used in over 50 published studies. Importantly, the combination of HepaRG cells with the CometChip offers an effective strategy for enabling detection of DNA damage induced by a wide range of genotoxic chemicals.

Through a collaboration among Inotiv, MIT (Laboratory of Dr. B. Engelward), and Health Canada/University of Ottawa (Laboratory of Dr. C. Yauk), work has previously been done to leverage metabolically competent HepaRG cells for multi‐endpoint genotoxicity testing with the objective of creating an in vitro substitute for rodent genetic toxicology testing. Previously, we have developed, qualified, and published a detailed step‐by‐step protocol for medium‐throughput comprehensive DNA damage assessment that combines mode‐of‐action based transcriptomics (using the TGx‐DDI biomarker), and CometChip by testing a small group of test chemicals (Buick et al. 2020, 2021; Owiti et al. 2022). These studies revealed a remarkable concordance between the HepaRG CometChip and transcriptomics, showing that they are highly complementary. Furthermore, by combining results from the CometChip assay and the TGx‐DDI transcriptomic biomarker, calls were 100% accurate in identifying genotoxic chemicals (Buick et al. 2021).

**FIGURE 1:**
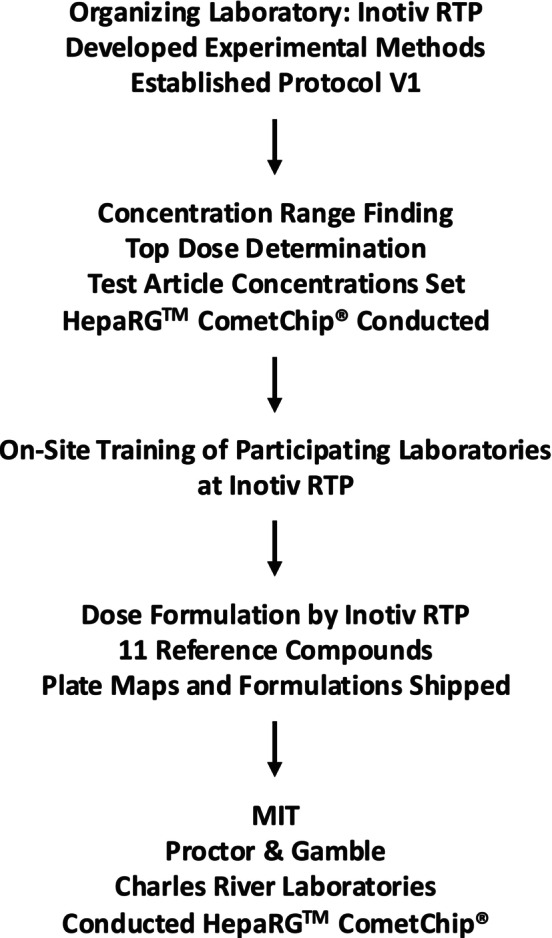
Programmatic overview of the HepaRG CometChip interlaboratory ring trial.

To further explore the HepaRG CometChip assay as an in vitro genetic toxicology test system and as a potential alternative to the in vivo rodent liver comet assay (OECD 2016a), we conducted an interlaboratory evaluation using a set of 11 test compounds (Figure 1). Four laboratories participated: Inotiv (led by Les Recio), the Massachusetts Institute of Technology (MIT; led by Bevin Engelward), Charles River Laboratories (CRL‐Skokie; led by Dan Roberts) and Proctor and Gamble (P&G; led by Stefan Pfuhler). These 11 test compounds were initially tested at Inotiv using HepaRG cells in a 2D format with cytotoxicity assessed by cellular ATP levels and DNA damage assessment using automated 96‐well CometChip technology. The primary purpose of this study was to assess the transferability of the HepaRG CometChip protocol established at Inotiv and to compare results across laboratories.

## 2 Materials and Methods

### 2.1 Ring Trial Design

**FIGURE 2:**
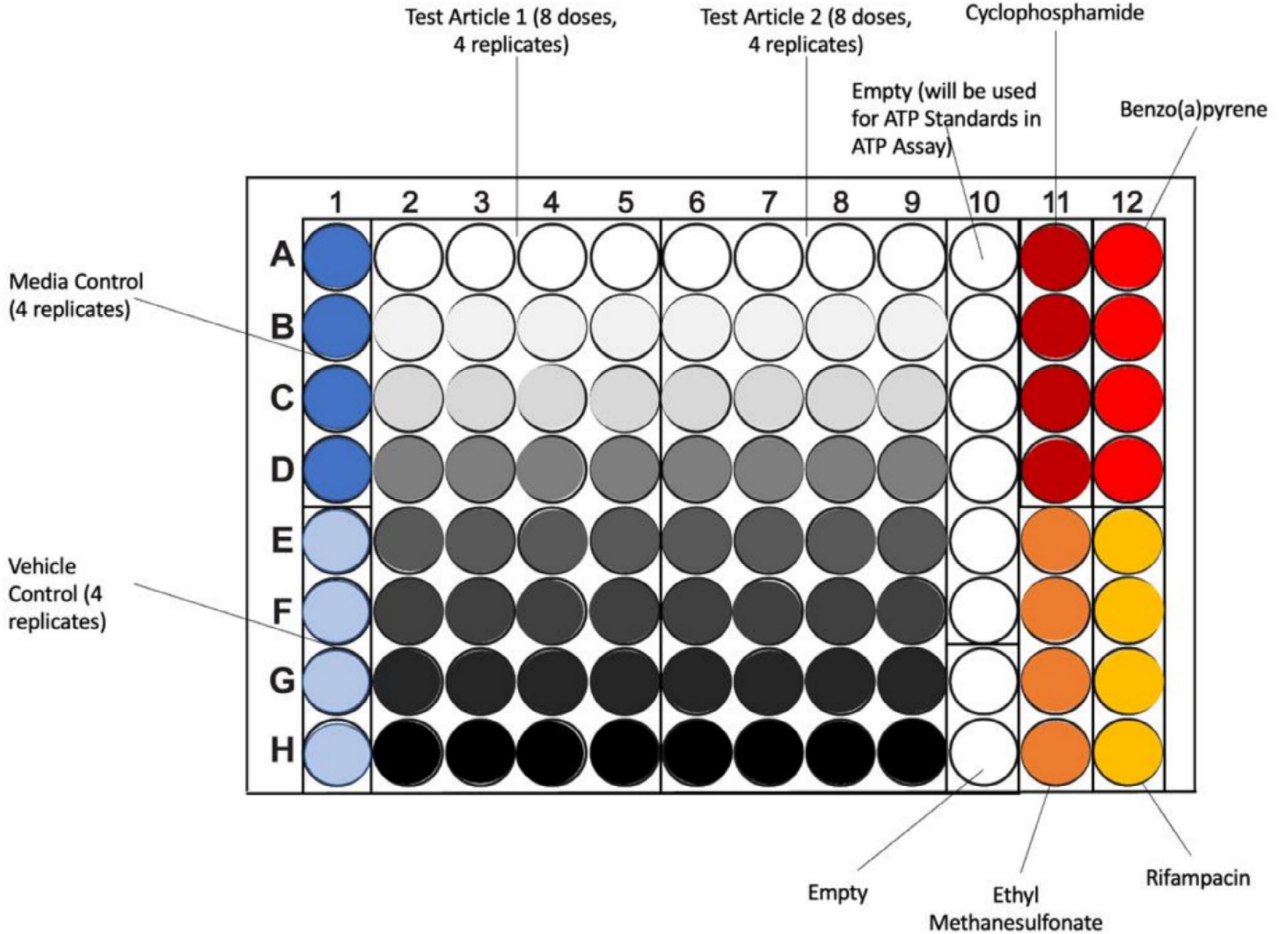
Standard plate map used during the interlaboratory evaluation of the HepaRG CometChip assay. Note that columns 11 and 12 were reference chemicals used for experiments not reported herein.

A 96‐well HepaRG CometChip assay with automated scoring using an identical set of 11 training compounds was evaluated across four independent laboratories (Figure 1). The project was organized by Inotiv who developed the methods and finalized the protocol in collaboration with the Engelward laboratory. After first establishing the protocol, completing dose‐range finding, and finishing testing of the 11‐compound set across at least two independent experiments, training of the other laboratories (P&G and CRL) was conducted on‐site at Inotiv using the published protocol (Owiti et al. 2022). The test compounds and highest test concentrations are shown in Table 1. Each compound was formulated in DMSO using serial dilutions and dispensed (and sealed) into 96‐well plates according to the plate map (Figure 2) prior to being shipped to participating laboratories on dry ice. For each agent, MIT ran at least two independent experiments while CRL and P&G conducted a single experiment, for which P&G evaluated compounds on the basis of two Comet Chips (technical replicates). Results were subsequently compiled and analyzed by Inotiv and MIT. Throughout these studies, there were regular meetings with Steering Committee members who had broad expertise relevant to the project.

**TABLE 1:** List of compounds tested and top test concentrations.

Compound	Abbreviation	CAS number	Top test concentration^a^
2‐acetylaminofluroene	2‐AAF	53‐96‐3	10 mM
Amitrole (3‐amino‐1,2,4‐triazole)	—	61‐82‐5	10 mM
Cadmium chloride	CAD	10108‐64‐2	20 μM
2,4‐dichlorophenol	DCP	120‐83‐2	500 μM
Eugenol	—	97‐53‐0	2 mM
Hydroquinone	HQ	123‐31‐9	1 mM
Azidothymidine	AZT	30516‐87‐1	2.7 mM
Benzo(a)pyrene	BP	50‐32‐8	25 μM
Cyclophosphamide	CP	50‐18‐0	10 mM
Ethyl methanesulfonate	EMS	62‐50‐0	10 mM
7,12‐dimethylbenzanthracene	DMBA	120‐83‐2	500 μM

*Note*: “—” indicates no abbreviation is used within.

^a^ Based on initial testing at Inotiv, with 10 mM being the default limit dose in the absence of cytotoxicity.

### 2.2 Chemical Selection

The 11 compounds were selected because they are well‐characterized, cover several different modes of action, and most have rodent comet data for comparison. Compounds were purchased from Millipore Sigma (St. Louis, MO, USA) and the vehicle used for testing was dimethyl sulfoxide (DMSO, 1% v/v). Six compounds with positive genotoxicity results in traditional regulatory tests were selected: ethyl methane sulfonate (EMS, an alkylating agent), azidothymidine (AZT, a nucleoside reverse transcriptase inhibitor), the polycyclic aromatic hydrocarbons benzo(a)pyrene (BP), 7,12‐dimethylbenz[a]anthracene (DMBA), 2‐aminoacetylfluorene (2‐AAF), and the cancer chemotherapeutic agent cyclophosphamide (CP, a pro drug). Five other compounds were selected as agents that are negative, induce genotoxicity secondarily, or have misleading positive test results in other genotoxicity assays. These were amitrole, cadmium chloride (CAD), hydroquinone (HQ), eugenol, and 2,4‐dichlorophenol (DCP).

### 2.3 HepaRG Cell Culture

Cryopreserved No‐Spin HepaRG cells were purchased from Lonza Biosciences (Triangle Research Labs, Durham, NC, USA). A detailed protocol for cell culture, chemical exposures, and conduct of the CometChip assay has been published and is illustrated in Figure S2 (Owiti et al. 2022; Buick et al. 2021). For the studies reported here, a 3‐day repeat exposure regimen was used as previously described (Buick et al. 2020, 2021; Owiti et al. 2022). Briefly, 80,000 cells were seeded in each well of a collagen‐coated 96‐well plate with Thawing and Plating Supplement. After 1 week of passaging, Pre‐induction/Tox Supplements were used during 3 daily chemical exposures, spaced ~24 h apart. Approximately 3–4 h after the final exposure initiation cells were harvested for cytotoxicity assessment and the CometChip assay.

### 2.4 Cytotoxicity Assessment

The CellTiter‐Glo Luminescent Cell Viability Assay (Promega, Madison, WI, USA) was used to determine the number of viable HepaRG cells based on the quantification of ATP. Cytotoxicity was evaluated following the manufacturer's instructions in 96‐well plates. Briefly, wells containing 100 μL cell samples were equilibrated at room temperature for 30 min prior to the addition of CellTiter‐Glo Reagent to each well in a volume equal to that of the cell culture medium (e.g., 100 μL). Some labs added the CellTiter‐Glo Reagent directly to exposure plates after sampling for the CometChip assay as 100 μL dead volume remained. The contents were mixed for 2 min on an orbital shaker to enhance cell lysis prior to incubation at room temperature for 10 min to stabilize the luminescent signal. Luminescence was measured on a plate reader (e.g., SpectraMax, Molecular Devices, San Jose, CA, USA) and is directly proportional to the number of viable cells in the culture. The relative survival cut‐off was 30% to assess DNA damage from test compound exposures (up to 70% chemical‐induced cytotoxicity). Cytotoxicity assessments for all chemicals were conducted at Inotiv to select concentrations for the interlaboratory ring trial prior to shipping dose formulations to participating laboratories. Non‐cytotoxic chemicals were tested up to 10 mM, which is in line with current regulatory guidelines for limit concentrations in cell‐based genotoxicity assays.

### 2.5 CometChip Assay

CometChips were obtained from Bio‐Techne (Gaithersburg, MD) and are presently available from CellArray LLC (Lexington, MA). Exposed and control HepaRG cells were trypsinized; 100 μL was loaded into CometChip wells using either the supplied manifold or a bottomless 96‐well plate. After 15–20 min at 37°C to allow cells to settle into CometChip pores (e.g., microwells that are 30 µM in diameter), a 1% agarose overlay was applied and cells were lysed in cold lysis buffer (2.5 M NaCl, 100 mM EDTA, 10 mM Tris, pH 10 with 1% Triton X‐100 [Sigma, St. Louis, MO, USA] plus 10% DMSO) for 2–24 h. Following lysis, the CometChip was equilibrated in an alkaline electrophoresis buffer (300 mM NaOH/1 mM EDTA) for 60 min and electrophoresed for 50 min under a 300 mA current at 4°C. These alkaline conditions detect multiple types of DNA damage including DNA single strand breaks. Following electrophoresis, the CometChips were neutralized at 4°C for 2 × 15 min in 0.4 M Tris, pH 7.4 and equilibrated overnight at 4°C in 20 mM Tris, pH 7.4. CometChips were then stained for 30 min at 4°C in 0.1X SYBR Gold and stored in 20 mM Tris, pH 7.4 for > 1 h. at 4°C as they awaited imaging. Images of all 96 wells were taken with a 4X objective and tiff files were analyzed using Trevigen Comet Analysis Software (version 1.3b). The Trevigen software automatically calculates percent tail DNA. Comet selection was done in an automated fashion without intervention by the experimentalist to ensure unbiased results.

### 2.6 CometChip Data Analysis

The image analysis software automatically calculated %DNA in Tail (“%Tail DNA” throughout) for each comet per well. Comet selection was done in an automated fashion without intervention by the experimentalist to ensure unbiased results. Due to inherent loading differences across each plate, median %Tail DNA was calculated for ≥ 50 cells per well with typical loading efficiency of ~200 to 400 cells per 96 well. The average of %Tail DNA was then calculated per exposure condition, with typically four wells exposed per concentration of test chemical. When only one experiment was conducted, this mean (across four wells) was presented with variation represented by standard deviation. When ≥ 2 independent experiments were conducted, the average across experiments is presented with variation displayed as standard error of the mean.

The %Tail DNA data were analyzed by Student's *t*‐test and one‐way ANOVA followed by Dunnett's test to correct for multiple comparisons when comparing treatment groups to the control group. Results were considered statistically significant when *p* < 0.05. Three criteria are commonly used to assess positive response across in vitro genotoxicity tests: (a) a statistically significant increase relative to vehicle control, (b) the response maintains a monotonic concentration‐related increase, and (c) the response exceeds historical biological variation in vehicle/negative controls. This latter point cannot be assessed until sufficient data are available, so the first two criteria were used. Specifically, in addition to pairwise significance, concentration related significance across two or more consecutive concentrations were considered to be positive, with the exception that in cases where the maximum % Tail DNA was below 40%, a result where % Tail DNA rose but then fell at higher doses were considered negative.

## 3 Results and Discussion

Each participating laboratory produced data for the 11 reference compounds that were deemed acceptable for interpretation. Results from only three experiments, out of the 44 conducted, were inconsistent, resulting in over 93% agreement in overall test results (Table 2). Of the 11 compounds tested, eight generated unanimous calls in all labs and three had the same call in three of the four labs. The following is a discussion of the interlaboratory HepaRG CometChip test results for each compound.

**TABLE 2:** HepaRG ComtChip results across laboratories.

Compound	Inotiv	P&G	CRL	MIT
2‐AAF	−	−	−	−
Amitrole	−	−	−	+
CAD	−	−	+	−
DCP	−	−	−	−
Eugenol	−	−	−	−
HQ	−	−	−	−
DMBA	−	−	−	+
AZT	+	+	+	+
BP	+	+	+	+
CP	+	+	+	+
EMS	+	+	+	+

*Note*: Overall test results from each participating laboratory.

Abbreviations: positive test result indicated by “+,” negative test result indicated by “−.”

### 3.1 Negative Compounds

#### 3.1.1 2‐Aminoacetylfluorene (2‐AAF)

**FIGURE 3:**
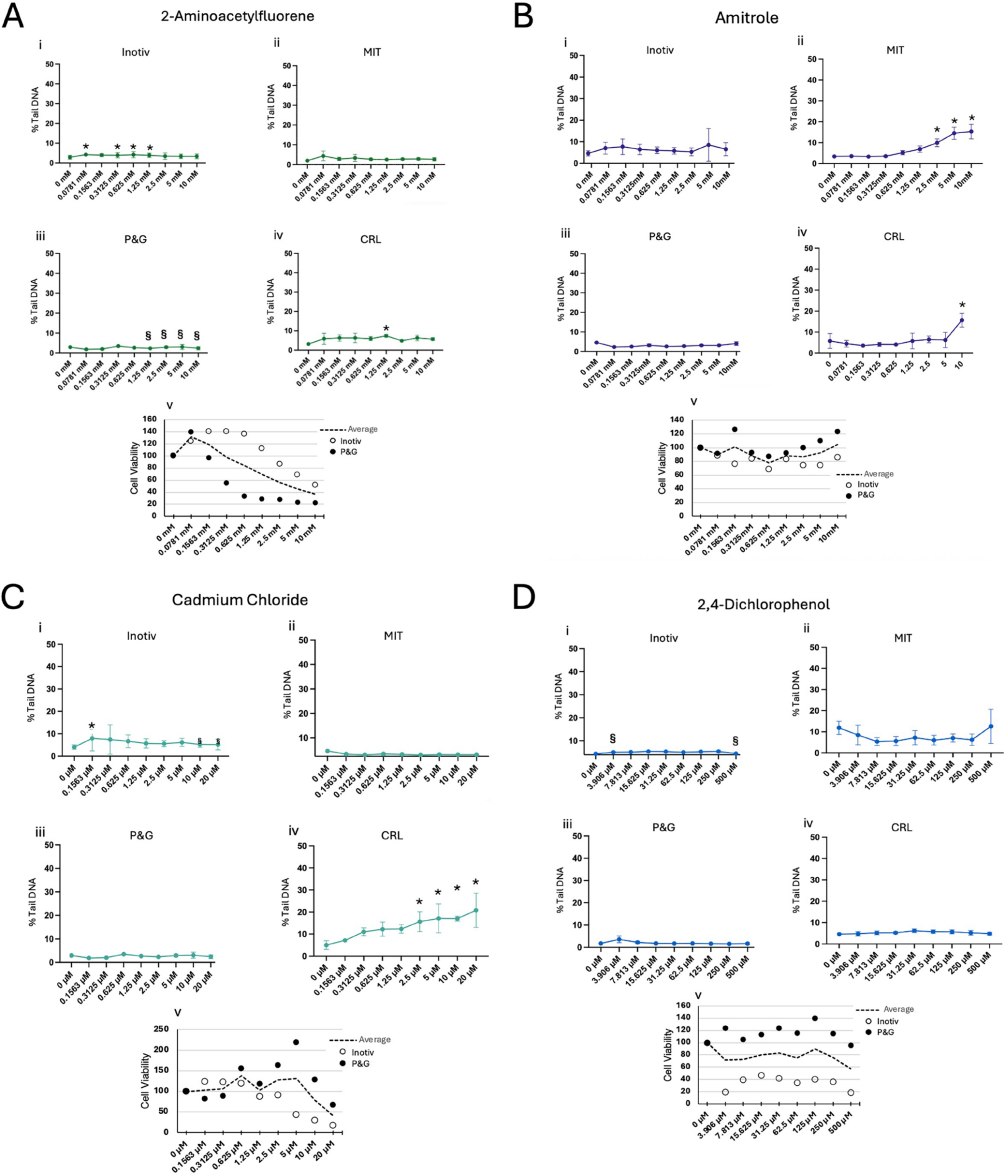
Results for 2‐aminoacetylfluorene (A), amitrole (B), cadmium chloride (C) and 2,4‐dichlorophenol (D). For all compounds: (i) three or more independent tests, experimental means ± SEM. (ii) Two or more independent tests, experimental means ± SEM. (iii) Single exposure, analyzed on duplicate CometChips, plate means ± SEM. (iv) Single test, plate means ± SD. (v) Cell viability results for Cell Titer‐Glo. *Statistical significance in at least one experiment, *p* ≤ 0.05. §Overt cytotoxicity, ≤ 30% relative survival (Cell TiterGlo).

Across all four laboratories, there were no concentration related increases in %Tail DNA up to 10 mM (Figure 3A). In the first lab, there was a statistically significant increases in the center of the dose response with no significant increase at three subsequent higher concentrations (2.5–10 mM, Figure 3Ai). Hence, these results did not meet the criteria for positive response. This inconsistent statistical significance mirrors early studies in rats, where 2‐AAF was considered “equivocal” in the alkaline liver comet assay (Uno and Omori 2015). However, repeat studies and review of the data by an expert working group suggest that 2‐AAF is genotoxic and mutagenic in liver (Kirkland et al. 2019). In previous studies with HepaRG cells exposed to 2‐AAF, results were positive in the comet assay with 3D spheroids but not in the 2D format, which is what was used here (Mandon et al. 2019). This could be due to differences in the efficiency of Phase 1 or 2 metabolism when grown as spheroids (Ramaiahgari et al. 2017).

Additionally, 2‐AAF creates bulky lesions that are repaired by nucleotide excision repair (NER). Bulky lesions do not impact DNA migration and so cannot themselves be detected, while single strand breaks created during repair are potentially detectable using the alkaline comet assay. However, unlike base excision repair intermediates that are readily detected using the alkaline comet assay, NER intermediates are not. A likely reason is because NER is much slower than BER. As such, NER intermediates are both transient and spread out over time, such that at any particular timepoint, levels are anticipated to be low and thus hard to detect.

#### 3.1.2 Amitrole

Amitrole was considered negative in three laboratories when tested up to 10 mM (Figure 3B). Conversely, concentration related increases in %Tail DNA were observed and reproduced in one laboratory with statistically significant responses observed at ≥ 2.5 mM (Figure 3Bii). This was unexpected and did not correlate with high cytotoxicity (Figure 3Bv). Overall, a negative call was concordant across three of four laboratories and Amitrole is generally considered non‐genotoxic despite inducing thyroid and liver tumors in rodents (via non‐DNA reactive mechanisms) (IARC 1987; Mattioli et al. 1994).

#### 3.1.3 Cadmium Chloride (CAD)

The majority of laboratories (three of four) found CAD negative in the HepaRG CometChip assay (Figure 3Ci–iii). One lab that conducted a single experiment generated concentration dependent increases in %Tail DNA at ≥ 2.5 mM (Figure 3Civ). Additional testing (confirmatory trials within this lab) would have been useful to investigate reproducibility of this response. Overall, the majority call (negative) is consistent with previous results using 2D HepaRG cultures (Seo et al. 2020). Interestingly, Seo and colleagues also showed that when exposing HepaRG 3D spheroids to CAD, positive results are obtained (Seo et al. 2020). The JaCVAM panel concluded that CAD was judged to be equivocal by OECD 489 criteria in the liver comet assay (Uno and Omori 2015; OECD 2016a). The mechanism of CAD induced genotoxicity appears pleiotropic, with an oxidative stress component, and is not related to direct DNA adduct formation (Boisvert et al. 2023; Reviewed in Hartwig et al. 2020).

#### 3.1.4 2,4‐Dichlorophenol (DCP)

DCP was negative in all laboratories when evaluated up to 500 μM (Figure 3D). DCP uncouples oxidative phosphorylation, which increases oxidative stress and leads to mixed in vitro genotoxicity test results but no carcinogenic activity in rodents (Hilliard et al. 1998; National Toxicology Program 1989). In vivo, DCP lacks genotoxicity, in part due to detoxification by enzymatic (e.g., catalase, superoxide dismutase) and nonenzymatic (e.g., glutathione) mechanisms. With regards to liver comet and the work presented here, activity of the NRF2/ARE defense network may also induce the expression of these antioxidant enzymes and Phase II detoxifying enzymes effectively protecting HepaRG cells from DNA damage (Allameh et al. 2023). This hypothesis is supported by data generated in a different genotoxicity NAM, ToxTracker, where the Nrf2 pathway in murine cells was activated (via increases in Srxn1), and no genotoxicity was detected (Hendriks et al. 2016). Hence, HepaRG, with more physiologically relevant detoxification activity, may increase specificity of cell‐based genotoxicity results as chromosomal damage has been noted in other test systems (Hilliard et al. 1998).

#### 3.1.5 Eugenol

**FIGURE 4:**
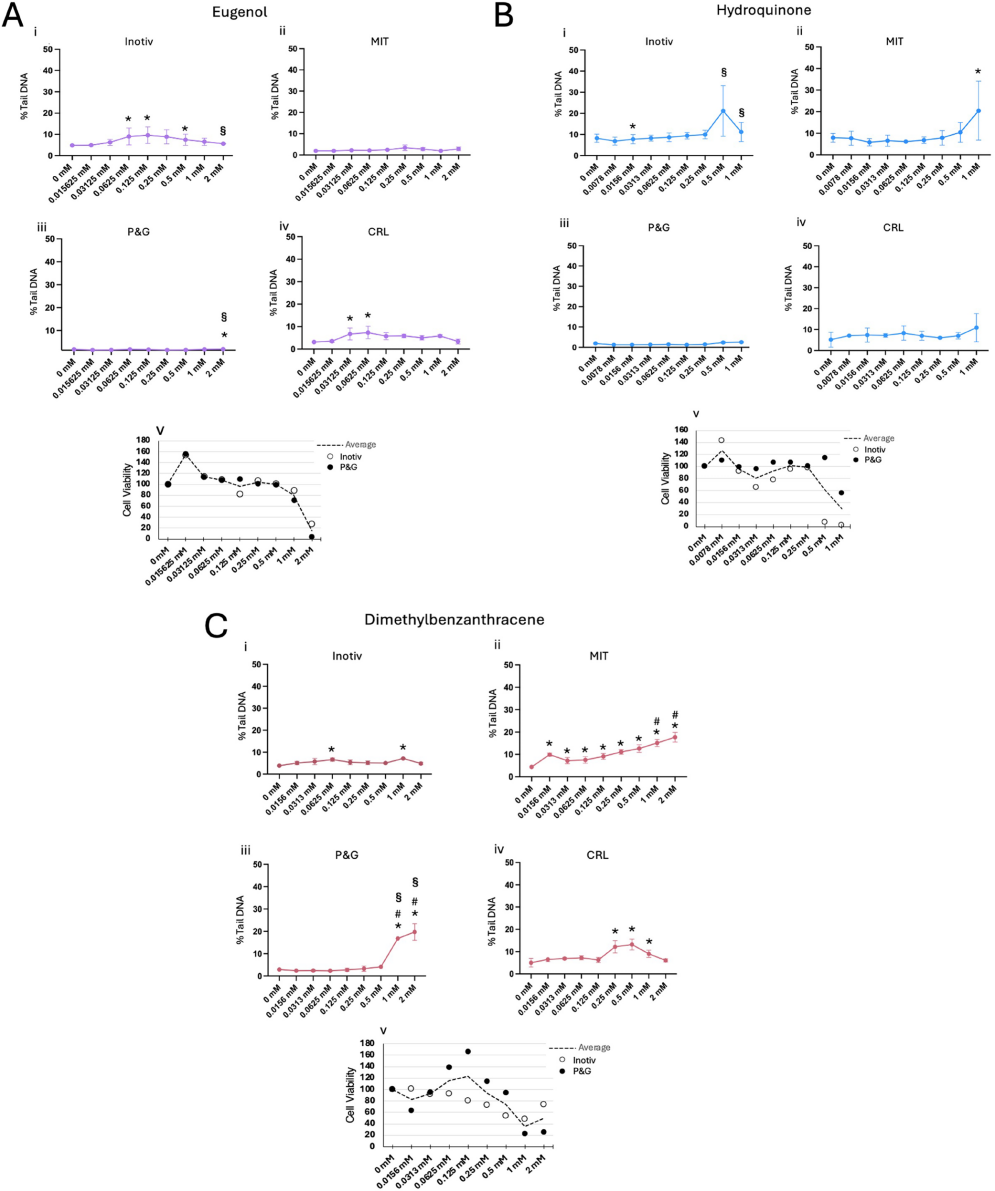
Results for eugenol (A), hydroquinone (B), and dimethylbenzanthracene (C). For all compounds: (i) Three or more independent tests, experimental means ± SEM. (ii) Two or more independent tests, experimental means ± SEM. (iii) Single exposure, analyzed on duplicate CometChips, plate means ± SEM. (iv) Single test, plate means ± SD. (v) Cell viability results for cell Titer‐Glo. *Statistical significance in at least one experiment, *p* ≤ 0.05. #Statistical significance across the average of all experiments. §Overt cytotoxicity, ≤ 30% relative survival (Cell Titer‐Glo).

Across all laboratories, eugenol did not induce %Tail DNA in a concentration related manner up to 2 mM with marked (~80%) cytotoxicity (Figure 4A). In two of the labs there were statistically significant increases early in the dose response curve that did not persist at higher exposures and was considered negative (Figure 4i,iv). Eugenol has produced a mixed set of genotoxic responses when evaluated in vitro but results herein were consistent with previous HepaRG CometChip studies (Buick et al. 2021). In another genotoxicity NAM, the turkey egg genotoxicity assay, which has functional metabolic capacity, eugenol did not induce DNA adducts or genotoxicity (Kobets et al. 2016). This compound is another good example of how HepaRG cells can enhance specificity compared to traditional apical endpoint assays, as results were not confounded by cytotoxicity.

#### 3.1.6 Hydroquinone (HQ)

HQ was negative in all four labs when evaluated up to 1 mM (Figure 4B). A statistically significant increase at 1 mM was observed in one lab, with a very high variance between experiments (Figure 4Bii). This effect may be driven by cytotoxicity, as ≤ 30% relative survival was observed by the organizing laboratory at ≥ 500 μM (Figure 4Bv). HQ, which induces oxidative stress, was comet negative in rats across multiple tissues including duodenum (a site of contact tissue), testes, liver, and kidneys (O'Donoghue et al. 2021). This primary mode of action (ROS induction) has been reported in other mechanistic investigations (Abokyi et al. 2021; Yang et al. 2023). Again the increased detoxification of HepaRG may be responsible for the predominantly negative CometChip test results.

#### 3.1.7 7,12‐Dimethylbenzanthracene (DMBA)

DMBA is positive in the rat liver comet assay (Shi et al. 2011) and induces bulky adducts after Phase 1 metabolism via epoxide formation (Todorovic et al. 1997). Herein, DMBA induced statistically significant results in a concentration related manner in a single lab (Figure 4Cii). For Inotiv and CRL, a few concentrations were significantly increased, but the increase was not monotonic and did not persist to the same or higher magnitude at the highest concentrations tested. Hence, results in these labs were considered negative (Figure 4Ci,iv). For P&G, %Tail DNA at the highest two concentrations were not considered significant as there was > 70% cytotoxicity in their lab resulting in a negative call up to 0.5 mM (Figure 4Ciii). Magnitudes of induced %Tail DNA were consistent with the observed results for 2‐AAF, as DMBA creates bulky lesions which are challenging to detect in the comet assay. Interpretation of CometChip results may be clarified with a more suitable cytotoxicity endpoint, which is described below (See Cytotoxicity Assay Remarks).

### 3.2 Positive Compounds

#### 3.2.1 Azidothymidine (AZT)

**FIGURE 5:**
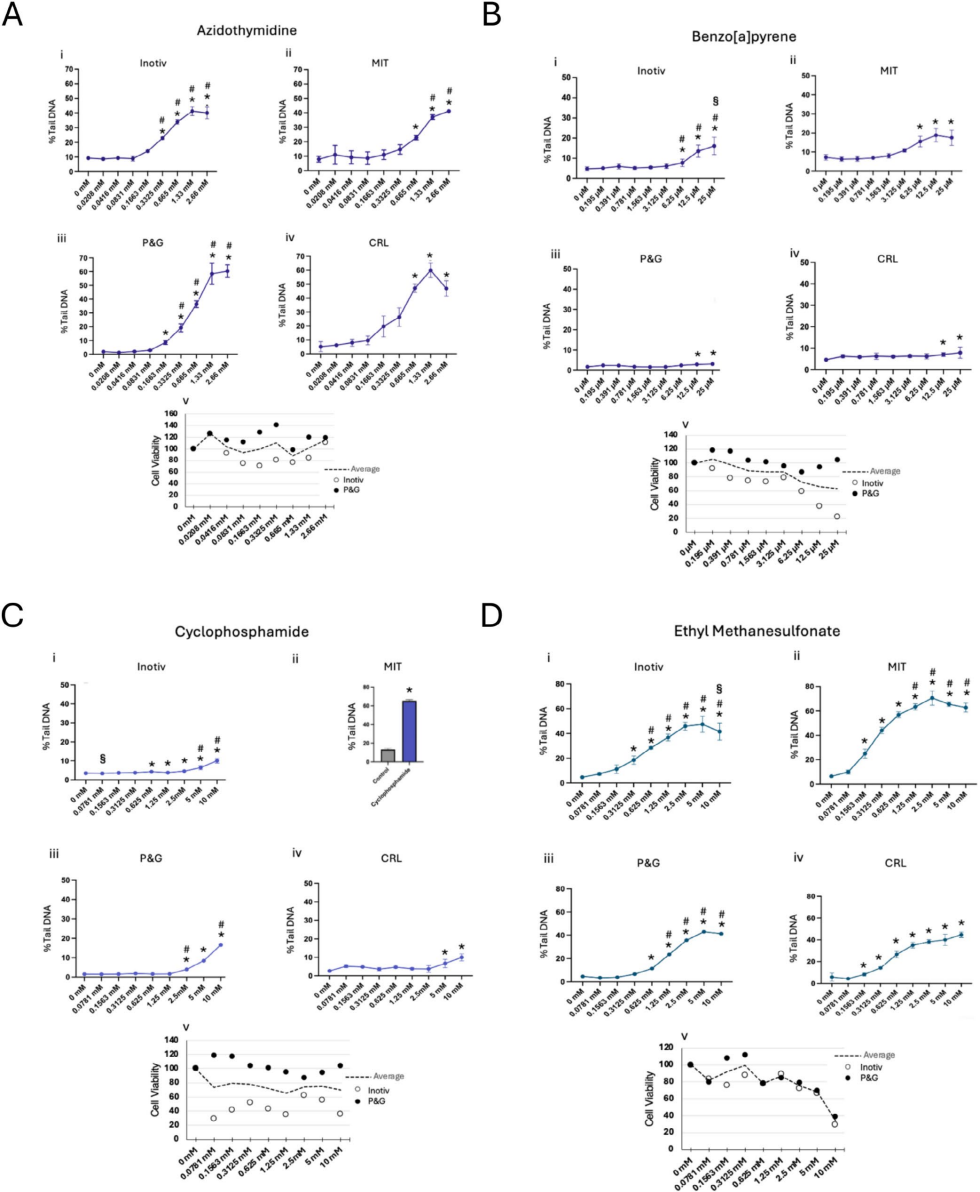
Results for azidothymidine (A), benzo(a)pyrene (B), cyclophosphamide (C) and ethyl methanesulfonate (D). For all compounds: (i) Three or more independent tests, experimental means ± SEM. (ii) Two or more independent tests, experimental means ± SEM. (iii) Single exposure, analyzed on duplicate CometChips, plate means ± SEM. (iv) Single experiment, plate means ± SD. *Statistical significance in at least one experiment, *p* ≤ 0.05. #Statistical significance across the average of all experiments. §Overt cytotoxicity, ≤ 30% relative survival (Cell Titer‐Glo).

There was a statistically significant dose related increase in %Tail DNA after AZT exposure in all four labs when evaluated up to 2.66 mM with no cytotoxicity (Figure 5A). Azidothymidine is a chain terminator during DNA replication and is genotoxic and mutagenic in rodents (Seier et al. 2012; Guérard et al. 2013). Inhibition of DNA replication is anticipated to lead to single strand gaps that are readily detected using the alkaline comet assay. There was excellent concordance among laboratories, regardless of the number of trials conducted at each test site, suggesting potential future use as a standard positive control in the assay.

#### 3.2.2 Benzo[a]Pyrene (BP)

BP induced statistically significant increases in %Tail DNA at ≥ 12.5 μM across all four labs (Figure 5B). The magnitude of the effect was low, which is likely due to the size of induced DNA adducts which are bulky and thus difficult to detect. In prior studies, after introducing an inhibitor of DNA replication (hydroxyurea and cytarabine [HU/AraC]), the %Tail DNA increased dramatically as NER intermediates had time to accumulate (Ngo et al. 2020). Hence, in the absence of HU/AraC a weak induction of %Tail DNA was expected. Finally, while BP is mutagenic in liver of MutaMouse, there are mixed reports of BP‐induced DNA damage in liver as detected by the comet assay in wild type rodents (Vaghef et al. 1996; Kirkland et al. 2019; Long et al. 2018).

#### 3.2.3 Cyclophosphamide (CP)

In all 4 laboratories CP induced statistically significant increases in %Tail DNA (Figure 5C). Clinically, CP is used in the treatment of leukemias/lymphomas and is inactive until metabolized by the liver, forming an adducting/crosslinking metabolite (i.e., a genotoxic prodrug). Efficacy as a chemotherapeutic agent for hematologic malignancies is likely due to spontaneous hydrolysis that occurs systemically when the circulating liver metabolite (namely, aldo phosphamide) creates a cytotoxic/genotoxic mustard compound and acrolein metabolites (Emadi et al. 2009). While it is genotoxic in peripheral tissues in vivo, CP is comet negative in the liver of rats (Recio et al. 2010). The lack of comets in liver is in part due to detoxification, but also CPs mechanism of action, as it forms DNA crosslinks, which inhibit rather than promote DNA migration and are thus not readily detected in the standard comet assay. Hence, the low induction of %Tail DNA herein when tested up to 10 mM, which is extreme for pharmaceuticals (generally limited to in vitro exposures of 1 mM, to avoid supraphysiological effects).

#### 3.2.4 Ethyl Methanesulfonate (EMS)

EMS is often used as a positive control when conducting in vivo comet assays (Recio et al. 2010, 2012; OECD 2016a). There were statistically significant dose related increases across all four labs at ≥ 0.625 mM (Figure 5D). Although the magnitude of the effect varied, dose responses are consistent among laboratories. EMS creates 7‐ethylguanine and 3‐ethyladenine, both of which may be repaired by base excision repair (BER). During BER, single strand breaks are requisite intermediates, and the comet assay is an effective approach to detect those repair intermediates, possibly because they occur in a concentrated fashion over a shorter period of time (compared to NER intermediates). Consistent with effective detection of DNA damage by EMS, other types of DNA damage repaired by BER likewise yield a strong signal in the CometChip assay (e.g., oxidative damage) (Owiti et al. 2022).

### 3.3 Cytotoxicity Assay Remarks

When setting up a 96‐well high throughput imaging method, standard cell counting procedures to determine increases in cell counts or relative population doubling are not easily integrated. Hence, ATP measurements were selected as the cytotoxicity endpoint and were demonstrably variable across laboratories, suggesting that an alternate method should be used with the CometChip technology. At the onset of this project, few genotoxicity publications with HepaRG existed, especially with a comet endpoint. Hence, the goal was to push exposures to a regulatory limit dose (i.e., 10 mM) or to limits of cytotoxicity (70% induced lethality), whichever was lower. This mimics current regulatory guidance for cell‐based genotoxicity assays and cytotoxicity limits used in other NAMs, which limit data interpretation to cultures with 50%–90% cell death (Bryce et al. 2017; Hendriks et al. 2016; OECD 2016b, 2023). The 70% cut off for cytotoxicity is higher than what is recommended for in vitro comet assays (30%), derived from analyses using standard test systems (i.e., tumor derived immortalized cell lines or primary white blood cells) that lack the endogenous detoxification pathways present in HepaRG cells (Azqueta et al. 2022). Although specificity of the assay increased when excluding concentrations that induced > 30%, HepaRG cells do not appear to yield false positive test results herein up to ~70% cytotoxicity. However, use of a more reproducible cytotoxicity endpoint would be needed to verify this observation.

## 4 Conclusions

This interlaboratory assessment of HepaRG CometChip was performed to evaluate the transferability and reproducibility of this NAM. There was 100% agreement in positive response among all four laboratories for four chemicals: EMS, AZT, BaP, and CP. Two chemicals that induce bulky DNA lesions (2‐AAF and DMBA) yielded negative or mixed results (respectively), which is consistent with prior work demonstrating that bulky lesions are difficult to detect using comet methodologies (Ngo et al. 2020). Additional sources of variability may have masked weak responses. For example, variation can be introduced due to differences in gel running tanks, differences in milliamps, or differences in the temperature of the running buffer. Also, variability between microscopes has been suggested by meta‐analysis of in vivo comet data (Dertinger et al. 2023). Finally, it is important to consider that the condition of the HepaRG cells shipped to each laboratory may not have been consistent. Since the cytotoxicity endpoint (ATP measurements) was variable and inconsistent between laboratories, alternate endpoints should be considered with this test system. For future HepaRG CometChip work, considering responses observed herein, EMS and AZT would be useful future positive controls and to ensure that the cells are metabolically competent, CP could also be used.

The remaining five compounds were either negative across all four labs (HQ, eugenol and DCP) or were negative in a majority (three of four) of labs (CAD and amitrole). Three of these compounds have limited or weak genotoxic potential reported in rodents (amitrole, eugenol, and DCP) and were negative across most labs herein. This highlights the potential benefit of using human cell‐based NAMs as rodent genotoxicity data may be contradictory to conclusions on carcinogenic potential (IARC 1985, 1987, 1999).

A significant limitation of the cell lines currently used in genetic toxicology is their deficiency in Phase II enzymes that conjugate endogenous water‐soluble molecules (sulfate, glucuronate, and glutathione) to xenobiotics to form substrates that can be eliminated. Herein, we have demonstrated that such metabolically competent cells (HepaRG cells) can be combined with CometChip technology as an effective tool for studying the DNA damaging potential of chemicals that undergo bioactivation to become genotoxic without the need to supplement with exogenous liver homogenate. Additionally, the detoxification that inherent Phase II metabolism offers is an advantage as it should produce data that is physiologically more relevant. The HepaRG CometChip could potentially be enhanced via incorporation of HU/AraC, or finding an alternative method to measure cytotoxicity, both of which require further investigation.

The genetic toxicology testing battery for pharmaceuticals requires follow‐up in vivo genotoxicity testing in rodents across two tissues when an in vitro positive test result is obtained. Generally, this is the liver comet assay and micronucleus endpoint measured in bone marrow‐derived cells (ICH 2011). The context of use goal for HepaRG CometChip could be to replace rodent‐based follow‐up tests and evaluate both endpoints in this human‐relevant biological test system. Several laboratories are developing NAMs with HepaRG cells for comprehensive genotoxicity assessments that include assessing DNA damage using CometChip technology, detecting micronuclei to assess chromosomal damage, and conducting toxicogenomics profiling to assess DNA damage response using the TGx‐DDI/GENOMARK biomarkers (Ates et al. 2018; Buick et al. 2020, 2021; Guo et al. 2024; Štampar and Žegura 2024; Froetschl et al. 2025). Metabolically competent human HepaRG cells are ideally suited as a human‐relevant cell model that can be used early in drug discovery to assess enzyme induction, hepatotoxicity, metabolite profiling, and as a follow‐up to positive results from regulatory in vitro genetic toxicology testing (Bernasconi et al. 2019; Guo et al. 2024; Štampar and Žegura 2024). Ultimately, HepaRG cells have the potential to reduce or replace our complete reliance on rodents for human risk assessments of genotoxic agents. In general, no assay or NAM will be perfect. As it exists today, a battery of tests is used to evaluate genotoxic potential, to ensure nothing is missed. Capitalizing on the combined advantages of HepaRG cells and the CometChip platform, this NAM may be a useful addition (or alternative) to the in vitro components of the genetic toxicology testing battery.

## Author Contributions

L.R. and B.P.E. conceived of the project, performed data analysis, and prepared the manuscript. L.R. was the leader for the ILS site and B.P.E. was the leader for the MIT site where they contributed to experimental oversight. S.P. was the leader for the Procter and Gamble site and contributed to experimental oversight, data analysis, and manuscript preparation. D.J.R. was the leader for the Charles River site and contributed to experimental oversight, data analysis, and manuscript preparation. L.F.S. played a leadership role in the project for the Charles River Laboratories site. C.S., L.M., E.R., E.P., S.K., and N.A.O. performed experiments and contributed to data analysis.

## Funding

This work was supported by the SBIR 2B grant awarded to Inotiv RTP 5U44ES024698‐05 and in kind from Proctor and Gamble Co. and Charles River Laboratories (Skokie, IL).

## Conflicts of Interest

Dr. Bevin Engelward is a co‐inventor on the CometChip patent.

## Supporting information


**Figure S1:** (A) The CometChip is an agarose gel with thousands of 30 μm diameter wells in agarose at the same depth. (B) Attaching a bottomless 96‐well plate or alternate manifold enables analysis of over 300 comets in a single macrowell. (C) Cells drop into the microwells by gravity. (D) Excess cells are removed by sheer force. (E,F) A layer of low melting point agarose traps the cells in the agarose array. Subsequently, the sample is processed using standard alkaline comet assay conditions. *Source*: Figure adapted with permission from a publication in *Nucleic Acids Research* (Ngo et al. 2020).
**Figure S2:** On Day 0, cells are loaded into the CometChip. On Day 7, chemicals are added to the 96‐well CometChip. On Day 9, cells are trypsinized so that they can be analyzed by CometChip. *Source*: Figure reproduced with permission from a publication in *Current Protocols* (Owiti et al. 2022).

## Data Availability

All raw data are available upon request.

## References

[em70037-bib-0001] Abokyi, S. , S. W. Shan , C. H. Lam , et al. 2021. “Targeting Lysosomes to Reverse Hydroquinone‐Induced Autophagy Defects and Oxidative Damage in Human Retinal Pigment Epithelial Cells.” International Journal of Molecular Sciences 22, no. 16: 9042. 10.3390/ijms22169042. 34445748 PMC8396439

[em70037-bib-0002] Allameh, A. , R. Niayesh‐Mehr , A. Aliarab , G. Sebastiani , and K. Pantopoulos . 2023. “Oxidative Stress in Liver Pathophysiology and Disease.” Antioxidants 12, no. 9: 1653. 10.3390/antiox12091653. 37759956 PMC10525124

[em70037-bib-0003] Andersen, M. E. , P. D. McMullen , M. B. Phillips , et al. 2019. “Developing Context Appropriate Toxicity Testing Approaches Using New Alternative Methods (NAMs).” ALTEX 36, no. 4: 523–534. 31664457 10.14573/altex.1906261

[em70037-bib-0004] Ates, G. , B. Mertens , A. Heymans , et al. 2018. “A Novel Genotoxin‐Specific qPCR Array Based on the Metabolically Competent Human HepaRG Cell Line as a Rapid and Reliable Tool for Improved in Vitro Hazard Assessment.” Archives of Toxicology 92, no. 4: 1593–1608. 10.1007/s00204-018-2172-5. 29411056

[em70037-bib-0005] Azqueta, A. , H. Stopper , B. Zegura , M. Dusinska , and P. Møller . 2022. “Do Cytotoxicity and Cell Death Cause False Positive Results in the in Vitro Comet Assay?” Mutation Research, Genetic Toxicology and Environmental Mutagenesis 881: 503520. 10.1016/j.mrgentox.2022.503520. 36031332

[em70037-bib-0006] Bernasconi, C. , O. Pelkonen , T. B. Andersson , et al. 2019. “Validation of in Vitro Methods for Human Cytochrome P450 Enzyme Induction: Outcome of a Multi‐Laboratory Study.” Toxicology In Vitro 60: 212–228. 10.1016/j.tiv.2019.05.019. 31158489 PMC6718736

[em70037-bib-0007] Boisvert, L. , R. Derr , T. Osterlund , G. Hendriks , and I. Brandsma . 2023. “Quantitative Interpretation of ToxTracker Dose–Response Data for Potency Comparisons and Mode‐Of‐Action Determination.” Environmental and Molecular Mutagenesis 64, no. 2: 132–143. 10.1002/em.22525. 36645179

[em70037-bib-0008] Bryce, S. M. , D. T. Bernacki , J. C. Bemis , et al. 2017. “Interlaboratory Evaluation of a Multiplexed High Information Content in Vitro Genotoxicity Assay.” Environmental and Molecular Mutagenesis 58, no. 3: 146–161. 10.1002/em.22083. 28370322 PMC5436310

[em70037-bib-0009] Buick, J. K. , A. Williams , R. Gagné , et al. 2020. “Flow Cytometric Micronucleus Assay and TGx‐DDI Transcriptomic Biomarker Analysis of Ten Genotoxic and Non‐Genotoxic Chemicals in Human HepaRG Cells.” Genes and Environment 42: 5. 10.1186/s41021-019-0139-2. 32042365 PMC7001283

[em70037-bib-0010] Buick, J. K. , A. Williams , M. J. Meier , et al. 2021. “A Modern Genotoxicity Testing Paradigm: Integration of the High‐Throughput CometChip and the TGx‐DDI Transcriptomic Biomarker in Human HepaRG Cell Cultures.” Frontiers in Public Health 9: 694834. 10.3389/fpubh.2021.694834. 34485225 PMC8416458

[em70037-bib-0011] Cho, E. , A. Allemang , M. Audebert , et al. 2022. “AOP Report: Development of an Adverse Outcome Pathway for Oxidative DNA Damage Leading to Mutations and Chromosomal Aberrations.” Environmental and Molecular Mutagenesis 63, no. 3: 118–134. 10.1002/em.22479. 35315142 PMC9322445

[em70037-bib-0012] Dertinger, S. D. , D. Li , C. Beevers , et al. 2023. “Assessing the Quality and Making Appropriate Use of Historical Negative Control Data: A Report of the International Workshop on Genotoxicity Testing (IWGT).” Environmental and Molecular Mutagenesis. 10.1002/em.22541. PMC10598234 37097075

[em70037-bib-0013] Devito, M. , P. Farrell , S. Hagiwara , et al. 2024. Value of Information Case Study: Human Health and Economic Trade‐offs Associated with the Timeliness, Uncertainty, and Costs of the Draft EPA Transcriptomic Assessment Product (ETAP). U.S. Environmental Protection Agency, EPA/600/X‐23/082. 10.23645/epacomptox.26093572. 39383270

[em70037-bib-0014] Emadi, A. , R. J. Jones , and R. A. Brodsky . 2009. “Cyclophosphamide and Cancer: Golden Anniversary.” Nature Reviews Clinical Oncology 6, no. 11: 638–647. 10.1038/nrclinonc.2009.146. 19786984

[em70037-bib-0015] Franzosa, J. A. , J. A. Bonzo , J. Jack , et al. 2021. “High‐Throughput Toxicogenomic Screening of Chemicals in the Environment Using Metabolically Competent Hepatic Cell Cultures.” npj Systems Biology and Applications 7, no. 1: 1–15. 10.1038/s41540-020-00166-2. 33504769 PMC7840683

[em70037-bib-0016] Froetschl, R. , J. C. Corton , H. Li , et al. 2025. “Consensus Findings of an International Workshops on Genotoxicity Testing Workshop on Using Transcriptomic Biomarkers to Predict Genotoxicity.” Environmental and Molecular Mutagenesis. 10.1002/em.22645. PMC12988028 39757731

[em70037-bib-0017] Guérard, M. , J. Koenig , M. Festag , et al. 2013. “Assessment of the Genotoxic Potential of Azidothymidine in the Comet, Micronucleus, and *Pig‐a* Assay.” Toxicological Sciences 135, no. 2: 309–316. 10.1093/toxsci/kft148. 23811826

[em70037-bib-0018] Guo, K. , and T. van den Beucken . 2024. “Advances in Drug‐Induced Liver Injury Research: In Vitro Models, Mechanisms, Omics and Gene Modulation Techniques.” Cell & Bioscience 14: 134. 10.1186/s13578-024-01317-2. 39488681 PMC11531151

[em70037-bib-0019] Guo, X. , H. Xu , and J. E. Seo . 2024. “Application of HepaRG Cells for Genotoxicity Assessment: A Review.” Journal of Environmental Science and Health, Part C: Environmental Toxicology and Carcinogenesis 42, no. 3: 214–237. 10.1080/26896583.2024.2331956. PMC12395423 38566478

[em70037-bib-0020] Gutmann, M. , E. Stimpfl , G. Langmann , H. Koudelka , B. Mir‐Karner , and B. Grasl‐Kraupp . 2023. “Differentiated and Non‐Differentiated HepaRG Cells: A Possible in Vitro Model System for Early Hepatocarcinogenesis and Non‐Genotoxic Carcinogens.” Toxicology Letters 390: 15–24. 10.1016/j.toxlet.2023.10.014. 37890683

[em70037-bib-0021] Harrill, A. , K. Carstens , N. Sipes , et al. 2024. Validation, Qualification, and Regulatory Acceptance of New Approach Methodologies‐A Report of the Interagency Coordinating Committee on the Validation of Alternative Methods (ICCVAM) Validation Workgroup (VWG). NIH. 40418713

[em70037-bib-0022] Harrill, J. A. , L. J. Everett , D. E. Haggard , et al. 2024. “Signature Analysis of High‐Throughput Transcriptomics Screening Data for Mechanistic Inference and Chemical Grouping.” Toxicological Sciences 202, no. 1: 103–122. 10.1093/toxsci/kfae108. 39177380 PMC12261139

[em70037-bib-0023] Hartwig, A. , M. Arand , B. Epe , et al. 2020. “Mode of Action‐Based Risk Assessment of Genotoxic Carcinogens.” Archives of Toxicology 94, no. 6: 1787–1877. 10.1007/s00204-020-02733-2. 32542409 PMC7303094

[em70037-bib-0024] Hendriks, G. , R. S. Derr , B. Misovic , B. Morolli , F. M. Calléja , and H. Vrieling . 2016. “The Extended ToxTracker Assay Discriminates Between Induction of DNA Damage, Oxidative Stress, and Protein Misfolding.” Toxicological Sciences 150, no. 1: 190–203. 10.1093/toxsci/kfv323. 26719371 PMC5009621

[em70037-bib-0025] Hilliard, C. A. , M. J. Armstrong , C. I. Bradt , R. B. Hill , S. K. Greenwood , and S. M. Galloway . 1998. “Chromosome Aberrations in Vitro Related to Cytotoxicity of Nonmutagenic Chemicals and Metabolic Poisons.” Environmental and Molecular Mutagenesis 31, no. 4: 316–326. 9654240

[em70037-bib-0026] IARC . 1985. Eugenol IARC Monographs on the Evaluation of Carcinogenic Risks to Humans, Vol. 36: Allyl Compounds, Aldehydes, Epoxides and Peroxides, 95–120. International Agency for Research on Cancer.

[em70037-bib-0027] IARC . 1999. Polychlorophenols and Their Sodium Salts, IARC Monographs on the Evaluation of Carcinogenic Risks to Humans. Vol. 71. International Agency for Research on Cancer. PMC7682291 10476472

[em70037-bib-0028] IARC Working Group on the Evaluation of Carcinogenic Risks to Humans . 1987. Overall Evaluations of Carcinogenicity: An Updating of IARC Monographs Volumes 1 to 42. International Agency for Research on Cancer. (IARC Monographs on the Evaluation of Carcinogenic Risks to Humans, No. Supplement 7.) Amitrole (Group 2B). 3482203

[em70037-bib-0029] International Conference on Harmonisation of Technical Requirements for Registration of Pharmaceuticals for Human Use . 2011. “ICH Harmonised Tripartite Guideline Guidance On Genotoxicity Testing And Data Interpretation For Pharmaceuticals Intended For Human Use S2(R1).”

[em70037-bib-0030] Karmaus, A. L. , K. Mansouri , To KT , et al. 2022. “Evaluation of Variability Across Rat Acute Oral Systemic Toxicity Studies.” Toxicological Sciences 188, no. 1: 34–47. 10.1093/toxsci/kfac042. 35426934 PMC9237992

[em70037-bib-0031] Kirkland, D. , D. D. Levy , M. J. LeBaron , et al. 2019. “A Comparison of Transgenic Rodent Mutation and in Vivo Comet Assay Responses for 91 Chemicals.” Mutation Research ‐ Genetic Toxicology and Environmental Mutagenesis 839: 21–35. 30744809 10.1016/j.mrgentox.2019.01.007 PMC6697155

[em70037-bib-0032] Kobets, T. , J. D. Duan , K. D. Brunnemann , S. Etter , B. Smith , and G. M. Williams . 2016. “Structure–Activity Relationships for DNA Damage by Alkenylbenzenes in Turkey Egg Fetal Liver.” Toxicological Sciences 150, no. 2: 301–311. 10.1093/toxsci/kfv322. 26719370

[em70037-bib-0033] Long, A. S. , J. W. Wills , D. Krolak , et al. 2018. “Benchmark Dose Analyses of Multiple Genetic Toxicity Endpoints Permit Robust, Cross‐Tissue Comparisons of MutaMouse Responses to Orally Delivered Benzo[a]Pyrene.” Archives of Toxicology 92, no. 2: 967–982. 10.1007/s00204-017-2099-2. 29177888 PMC5818629

[em70037-bib-0034] Lübberstedt, M. , U. Müller‐Vieira , M. Mayer , et al. 2011. “HepaRG Human Hepatic Cell Line Utility as a Surrogate for Primary Human Hepatocytes in Drug Metabolism Assessment in Vitro.” Journal of Pharmacological and Toxicological Methods 63, no. 1: 59–68. 10.1016/j.vascn.2010.04.013. 20460162

[em70037-bib-0035] Mandon, M. , S. Huet , E. Dubreil , V. Fessard , and L. le Hégarat . 2019. “Three‐Dimensional HepaRG Spheroids as a Liver Model to Study Human Genotoxicity *in Vitro* With the Single Cell Gel Electrophoresis Assay.” Scientific Reports 9: 10548. 10.1038/s41598-019-47114-7. 31332230 PMC6646340

[em70037-bib-0036] Mattioli, F. , L. Robbiano , L. Fazzuoli , and P. Baracchini . 1994. “Studies on the Mechanism of the Carcinogenic Activity of Amitrole.” Fundamental and Applied Toxicology: Official Journal of the Society of Toxicology 23, no. 1: 101–106. 10.1006/faat.1994.1085. 7958553

[em70037-bib-0037] Moreau, M. , J. Fisher , M. E. Andersen , et al. 2022. “NAM‐Based Prediction of Point‐Of‐Contact Toxicity in the Lung: A Case Example With 1,3‐Dichloropropene.” Toxicology 481: 153340. 10.1016/j.tox.2022.153340. 36183849

[em70037-bib-0038] Najjar, A. , A. Punt , J. Wambaugh , et al. 2022. “Towards Best Use and Regulatory Acceptance of Generic Physiologically Based Kinetic (PBK) Models for In Vitro‐to‐In Vivo Extrapolation (IVIVE) in Chemical Risk Assessment.” Archives of Toxicology 96, no. 12: 3407–3419. 36063173 10.1007/s00204-022-03356-5 PMC9584981

[em70037-bib-0039] National Academies of Sciences, Engineering, and Medicine . 2022. New Approach Methods (NAMs) for Human Health Risk Assessment: Proceedings of a Workshop–in Brief. National Academies Press.

[em70037-bib-0040] National Academies of Sciences, Engineering, and Medicine . 2023. Building Confidence in New Evidence Streams for Human Health Risk Assessment: Lessons Learned From Laboratory Mammalian Toxicity Tests. National Academies Press. 37579039

[em70037-bib-0041] National Toxicology Program . 1989. “NTP Toxicology and Carcinogenesis Studies of 2,4‐Dichlorophenol (CAS No. 120‐83‐2) in F344/N Rats and B6C3F1 Mice (Feed Studies).” National Toxicology Program Technical Report Series 353: 1–182. 12704431

[em70037-bib-0042] Ngo, L. P. , N. A. Owiti , C. Swartz , et al. 2020. “Sensitive CometChip Assay for Screening Potentially Carcinogenic DNA Adducts by Trapping DNA Repair Intermediates.” Nucleic Acids Research 48, no. 3: e13. 10.1093/nar/gkz1077. 31822921 PMC7026589

[em70037-bib-0043] O'Donoghue, J. L. , C. Beevers , and A. Buard . 2021. “Hvdroquinone: Assessment of Genotoxic Potential in the *in Vivo* Alkaline Comet Assay.” Toxicology Reports 8: 206–214. 10.1016/j.toxrep.2021.01.005. 33489780 PMC7810912

[em70037-bib-0044] OECD . 2016a. Test No. 489: In Vivo Mammalian Alkaline Comet Assay, OECD Guidelines for the Testing of Chemicals, Section 4. OECD Publishing. 10.1787/9789264264885-en.

[em70037-bib-0045] OECD . 2016b. Test No. 490: In Vitro Mammalian Cell Gene Mutation Tests Using the Thymidine Kinase Gene, OECD Guidelines for the Testing of Chemicals, Section 4. OECD Publishing. 10.1787/9789264264908-en.

[em70037-bib-0046] OECD . 2023. Test No. 487: In Vitro Mammalian Cell Micronucleus Test, OECD Guidelines for the Testing of Chemicals, Section 4. OECD Publishing. 10.1787/9789264264861-en.

[em70037-bib-0047] Owiti, N. A. , S. Kaushal , L. Martin , et al. 2022. “Using the HepaCometChip Assay for Broad‐Spectrum DNA Damage Analysis.” Current Protocols 2, no. 9: e563. 10.1002/cpz1.563. 36165707 PMC9522315

[em70037-bib-0048] Paudel, I. , A. R. Barutcu , R. Samuel , et al. 2023. “Increasing Confidence in New Approach Methodologies for Inhalation Risk Assessment With Multiple End Point Assays Using 5‐Day Repeated Exposure to 1,3‐Dichloropropene.” Toxicology 499: 153642. 10.1016/j.tox.2023.153642. 37863466

[em70037-bib-0049] Ramaiahgari, S. C. , S. Waidyanatha , D. Dixon , M. J. DeVito , R. S. Paules , and S. S. Ferguson . 2017. “From the Cover: Three‐Dimensional (3D) HepaRG Spheroid Model With Physiologically Relevant Xenobiotic Metabolism Competence and Hepatocyte Functionality for Liver Toxicity Screening.” Toxicological Sciences 159, no. 1: 124–136. 10.1093/toxsci/kfx122. 28633424 PMC5837526

[em70037-bib-0050] Recio, L. , J. Fowler , L. Martin , and C. Swartz . 2023. “Genotoxicity Assessment in HepaRG Cells as a New Approach Methodology Follow up to a Positive Response in the Human TK6 Cell Micronucleus Assay: Naphthalene Case Study.” Environmental and Molecular Mutagenesis 64, no. 8‐9: 458–465. 10.1002/em.22575. 37704589

[em70037-bib-0051] Recio, L. , C. Hobbs , W. Caspary , and K. L. Witt . 2010. “Dose–Response Assessment of Four Genotoxic Chemicals in a Combined Mouse and Rat Micronucleus (MN) and Comet Assay Protocol.” Journal of Toxicological Sciences 35, no. 2: 149–162. 10.2131/jts.35.149. 20371966 PMC3520611

[em70037-bib-0052] Recio, L. , G. E. Kissling , C. A. Hobbs , and K. L. Witt . 2012. “Comparison of Comet Assay Dose–Response for Ethyl Methanesulfonate Using Freshly Prepared Versus Cryopreserved Tissues.” Environmental and Molecular Mutagenesis 53, no. 2: 101–113. 10.1002/em.20694. 22069077

[em70037-bib-0053] Seier, T. , G. Zilberberg , D. M. Zeiger , and S. T. Lovett . 2012. “Azidothymidine and Other Chain Terminators Are Mutagenic for Template‐Switch‐Generated Genetic Mutations.” Proceedings of the National Academy of Sciences of the United States of America 109, no. 16: 6171–6174. 10.1073/pnas.1116160109. 22474374 PMC3341039

[em70037-bib-0054] Seo, J. E. , Q. Wu , M. Bryant , et al. 2020. “Performance of High‐Throughput CometChip Assay Using Primary Human Hepatocytes: A Comparison of DNA Damage Responses With in Vitro Human Hepatoma Cell Lines.” Archives of Toxicology 94, no. 6: 2207–2224. 10.1007/s00204-020-02736-z. 32318794

[em70037-bib-0055] Sewell, F. , C. Alexander‐White , S. Brescia , et al. 2024. “New Approach Methodologies (NAMs): Identifying and Overcoming Hurdles to Accelerated Adoption.” Toxicological Research (Cambridge) 13, no. 2: tfae044. 10.1093/toxres/tfae044. PMC10964841 38533179

[em70037-bib-0056] Shao, K. , Q. Chen , and Z. Wang . 2019. “Quantifying Association Between Liver Tumor Incidence and Early‐Stage Liver Weight Increase—An NTP Data Analysis.” Toxicology Reports 6: 674–682. 10.1016/j.toxrep.2019.07.001. 31360640 PMC6639686

[em70037-bib-0057] Shi, J. , L. Krsmanovic , S. Bruce , et al. 2011. “Assessment of Genotoxicity Induced by 7,12‐Dimethylbenz(a)anthracene or Diethylnitrosamine in the Pig‐a, Micronucleus and Comet Assays Integrated Into 28‐Day Repeat Dose Studies.” Environmental and Molecular Mutagenesis 52, no. 9: 711–720. 10.1002/em.20678. 21976072

[em70037-bib-0058] Štampar, M. , and B. Žegura . 2024. “In Vitro Hepatic 3D Cell Models and Their Application in Genetic Toxicology: A Systematic Review.” Mutation Research, Genetic Toxicology and Environmental Mutagenesis 900: 503835. 10.1016/j.mrgentox.2024.503835. 39617595

[em70037-bib-0059] Stucki, A. O. , T. S. Barton‐Maclaren , Y. Bhuller , et al. 2022. “Use of New Approach Methodologies (NAMs) to Meet Regulatory Requirements for the Assessment of Industrial Chemicals and Pesticides for Effects on Human Health.” Frontiers in Toxicology 4: 964553. 10.3389/ftox.2022.964553. 36119357 PMC9475191

[em70037-bib-0060] Todorovic, R. , F. Ariese , P. Devanesan , et al. 1997. “Determination of Benzo[a]Pyrene‐ and 7,12‐Dimethylbenz[a]Anthracene‐DNA Adducts Formed in Rat Mammary Glands.” Chemical Research in Toxicology 10, no. 9: 941–947. 10.1021/tx970003y. 9305574

[em70037-bib-0061] Uno, Y. , and T. Omori . 2015. “Re‐Analysis Results Using Medians of the Data From the JaCVAM‐Organized International Validation Study of the in Vivo Rat Alkaline Comet Assay.” Mutation Research, Genetic Toxicology and Environmental Mutagenesis 786‐788: 182–187. 10.1016/j.mrgentox.2015.06.005. 26212310

[em70037-bib-0062] Vaghef, H. , A. C. Wisén , and B. Hellman . 1996. “Demonstration of Benzo(a)pyrene‐Induced DNA Damage in Mice by Alkaline Single Cell Gel Electrophoresis: Evidence for Strand Breaks in Liver but Not in Lymphocytes and Bone Marrow.” Pharmacology & Toxicology 78, no. 1: 37–43. 10.1111/j.1600-0773.1996.tb00177.x. 8685085

[em70037-bib-0063] Wang, Z. , G. W. Walker , D. C. G. Muir , and K. Nagatani‐Yoshida . 2020. “Toward a Global Understanding of Chemical Pollution: A First Comprehensive Analysis of National and Regional Chemical Inventories.” Environmental Science & Technology 54: 2575–2584. 31968937 10.1021/acs.est.9b06379

[em70037-bib-0064] Wood, D. K. , D. M. Weingeist , S. N. Bhatia , and B. P. Engelward . 2010. “Single Cell Trapping and DNA Damage Analysis Using Microwell Arrays.” Proceedings of the National Academy of Sciences of the United States of America 107, no. 22: 10008–10013. 10.1073/pnas.1004056107. 20534572 PMC2890454

[em70037-bib-0065] Yang, X. , S. Dong , C. Li , et al. 2023. “Hydroquinone Triggers Pyroptosis and Endoplasmic Reticulum Stress via AhR‐Regulated Oxidative Stress in Human Lymphocytes.” Toxicology Letters 376: 39–50. 10.1016/j.toxlet.2023.01.005. 36646296

