# Supplementary material for: Transferability and Reproducibility of the HepaRG CometChip Assay
Source: Environ Mol Mutagen. 2025 Nov 26;66(9):478–91. doi: 10.1002/em.70037 (PMC12703573; doi:10.1002/em.70037)
Supplement: Supplementary file 1 — Figure S1: (A) The CometChip is an agarose gel with thousands of 30 μm diameter wells in agarose at the same depth. (B) Attaching a bottomless 96‐well plate or alternate manifold enables analysis of over 300 comets in a single macrowell. (C) Cells drop into the microwells by gravity. (D) Excess cells are removed by sheer force. (E,F) A layer of low melting point agarose traps the cells in the agarose array. Subsequently, the sample is processed using standard alkaline comet assay conditions. Source: Figure adapted with permission from a publication in Nucleic Acids Research (Ngo et al. 2020). Figure S2: On Day 0, cells are loaded into the CometChip. On Day 7, chemicals are added to the 96‐well CometChip. On Day 9, cells are trypsinized so that they can be analyzed by CometChip. Source: Figure reproduced with permission from a publication in Current Protocols (Owiti et al. 2022). [file EM-66-478-s001.pptx]

## Slide 1
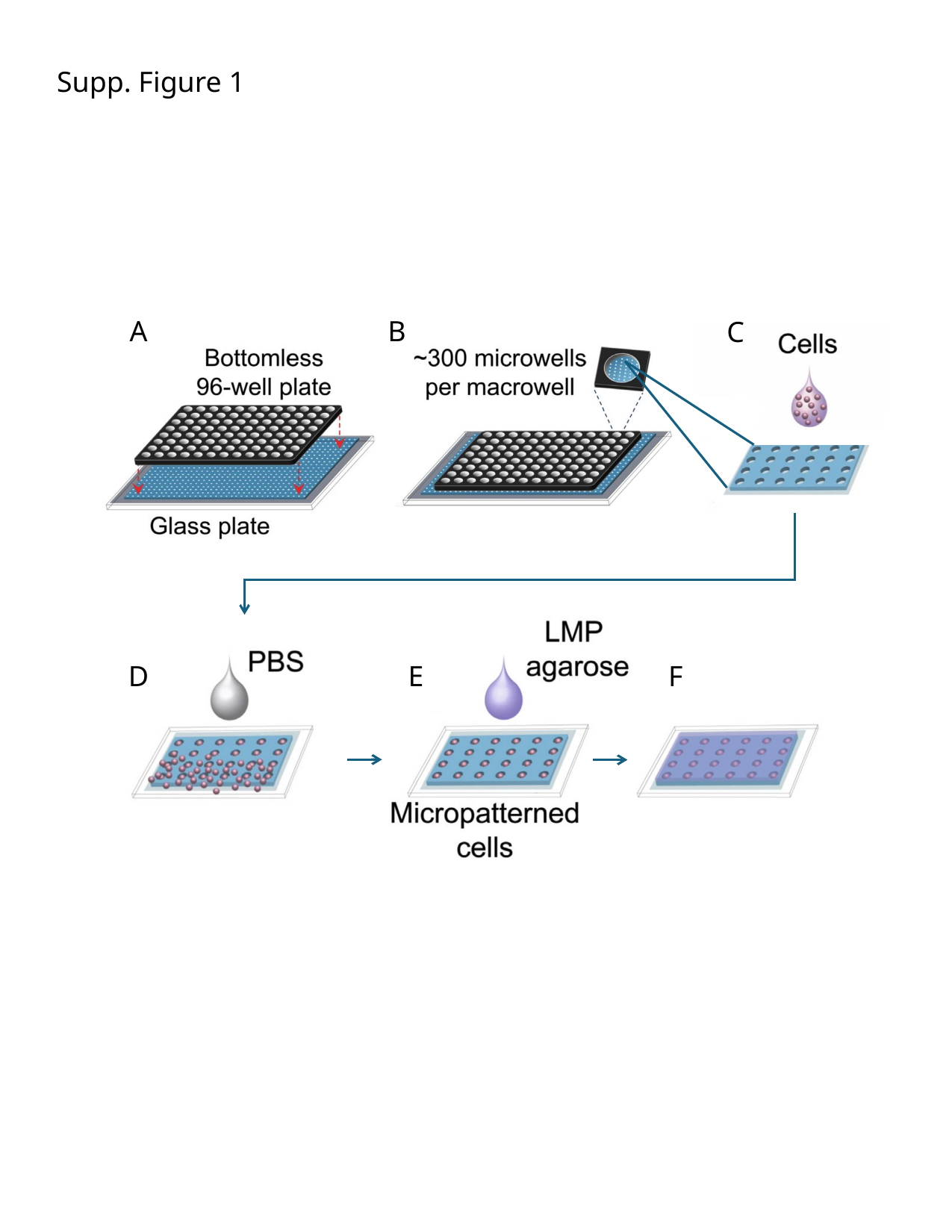

Supp. Figure 1
A
B
C
D
E
F

## Slide 2
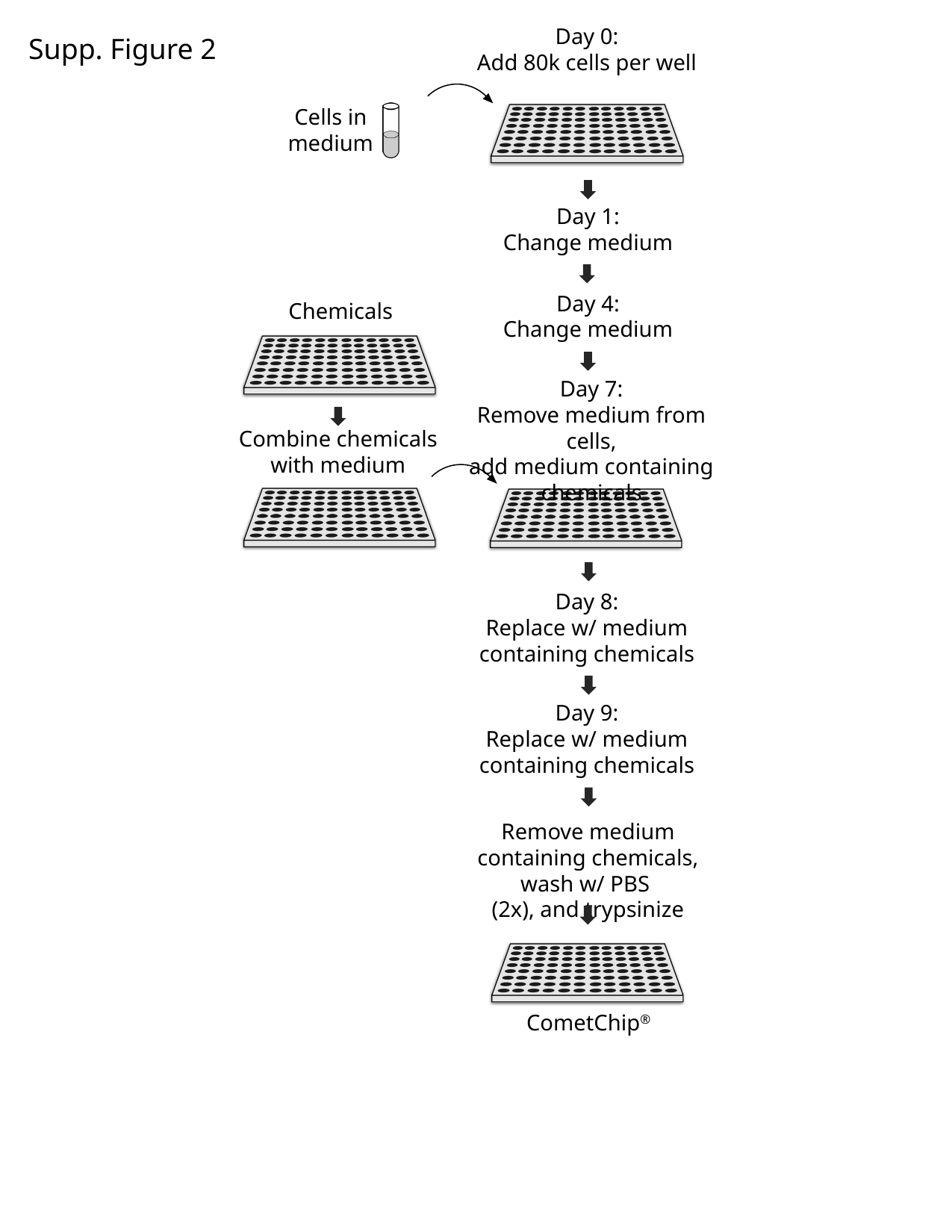

Day 0:
Add 80k cells per well
Supp. Figure 2
Cells in medium
Day 1:
Change medium
Day 4:
Change medium
Chemicals
Day 7:
Remove medium from cells,
add medium containing chemicals
Combine chemicals with medium
Day 8:
Replace w/ medium containing chemicals
Day 9:
Replace w/ medium containing chemicals
Remove medium containing chemicals, wash w/ PBS
(2x), and trypsinize
CometChip®
